# From classrooms to controllers: how school closures shaped children's video gaming habits

**DOI:** 10.1007/s00127-024-02635-z

**Published:** 2024-03-12

**Authors:** Muna Abed Alah, Sami Abdeen, Iheb Bougmiza, Nagah Selim

**Affiliations:** 1 Community Medicine Department, Hamad Medical Corporation (HMC), Doha, Qatar; 2 Community Medicine Department, Primary Health Care Corporation (PHCC), Doha, Qatar; 3 Community Medicine Department, College of Medicine, Sousse University, Sousse, Tunisia; 4 Community Medicine Department, Primary Health Care Corporation (PHCC), Doha, Qatar; 5 Public Health and Preventive Medicine, Cairo University, Cairo, Egypt; 6 Clinical Effectiveness Department, Primary Health Care Corporation, Doha, Qatar

**Keywords:** Video gaming, Internet gaming disorder, School closures, Screen time, Children

## Abstract

**Objectives:**

This study aimed to assess the impact of COVID-19-related school closures on screen time and video gaming habits among governmental school students in Qatar and explore the prevalence of Internet Gaming Disorder (IGD) within this context.

**Methods:**

A cross-sectional approach was employed, spanning two months from June to August 2022. A random sample of students aged 8–15 years was drawn from the national electronic health record system of Qatar. Telephone interviews with parents were conducted to collect data. The Parental Internet Gaming Disorder Scale (PIGDS) was used for IGD assessment.

**Results:**

Of 428 parents, 257 (60%) confirmed their child's engagement in video gaming during school closures. Participants averaged 11 years in age with 92 (35.8%) females and 165 (64.2%) males. Nationality included 62.6% expatriates and 37.4% Qatari locals. Average weekly screen time increased significantly from 19.7 ± 10.1 h to 31.9 ± 12.6 h during closure (*p* < 0.001). Video gaming time rose from 8.6 ± 8.6 h to 13.0 ± 12.4 h per week (*p* < 0.001). The prevalence of IGD was 8.6% (95% CI 5.4–12.7). Male students, expatriates, and those reporting increased video gaming time were more likely to develop IGD than their female and local counterparts.

**Conclusion:**

The observed associations between video gaming increase and IGD highlight the need for focused interventions to address potential risks and promote healthier digital habits among this population.

**Supplementary Information:**

The online version contains supplementary material available at 10.1007/s00127-024-02635-z.

## Introduction

Numerous countries worldwide have implemented various measures to curb the spread of COVID-19, such as the closure of malls, gyms, and schools [1, 2]. Qatar, for example, swiftly imposed protective measures that included the closure of schools in March 2020 [3]. While these measures have been successful in reducing the exponential transmission of the virus, their indirect impact on health and lifestyle cannot be overlooked. Although children and adolescents have been the least affected by the infection in terms of mortality and morbidity, they have not been immune to the adverse effects of home confinement measures and school closures on their lifestyle [4, 5]. Extensive research has demonstrated a decrease in physical activity, and an increase in sedentary behaviors and screen time among adolescents during periods of school closures [6–10]. Furthermore, the significant increase in the use of digital devices and screen time with the introduction of home confinement measures, either for leisure or as part of the online learning phenomenon, has resulted in a deterioration of eyesight among children and adolescents and an increase in the prevalence of digital eye strain or computer vision syndrome [11].

To cope with the stress surrounding the pandemic, children and adolescents have turned to online gaming as means of stress relief and escapism, leading to an increase in gaming behavior during the pandemic[12]. Internet gaming disorder (IGD) is becoming a growing concern for public health as it can have long-term, severe consequences on affected individuals, especially adolescents and young people. These consequences include self-esteem problems, emotional distress, impaired executive control, cognitive function, and disrupted regional structural connectivity[13]. The American Psychiatric Association (APA) has included IGD in the Diagnostic and Statistical Manual of Mental Disorders 5 (DSM-5) appendix as a potential diagnosis, with diagnostic criteria drafted based on substance use disorders [14, 15]. These criteria include preoccupation, withdrawal, tolerance, inability to reduce or stop gaming, disregard for other activities, continued gaming despite negative consequences, deception, using gaming as a means of escaping reality, and the potential risk to relationships due to excessive gaming [15]. A diagnosis of IGD can be made if an individual experiences five or more of these symptoms within a year [15]. The DSM-5 acknowledges that IGD may involve non-internet computerized games as well, but these games have been less researched than internet games. Therefore, if the diagnostic criteria are met, offline computerized games can be included in the definition of IGD [16]. A recent systematic review reported an increase in game usage time and game addiction score during the COVID-19 pandemic among children and adolescents in majority of studies [12]. Additionally, a new term, “videogame vision syndrome,” has been proposed to address vision issues related to prolonged screen use for playing videogames [17]. A retrospective study in Hong Kong reported that 83% of children and adolescents played videogames during the pandemic, with one-fifth of them being excessive gamers [18].

To the best of our knowledge, studies conducted in the Middle East to address the gaming behaviors of children and adolescents during COVID-19-related school closures are extremely limited. This study aimed to bridge this gap by exploring the gaming behaviors of children and adolescents and the prevalence of IGD during the period of school closures from parents’ perspective in Qatar and address the associated factors.

## Methods

In this cross-sectional study, the data collection phase was carried out over a period of two months, commencing in June and concluding in August 2022 targeting governmental schools’ students. A random sampling approach was employed to select participants from a sampling frame derived from the national electronic health record system of Qatar of students aged 8–15 years with the assistance of health information management team who provided us with the contact details of parents of selected students. In cases where children had identical father and family names, indicating they belonged to the same household, the health information management team was instructed to retain only one child's data (selected randomly) in the sampling frame provided for us. The data collection process involved conducting telephone interviews with the parents of the selected students. Arabic- or English-speaking parents were included. The commencement of the interview was marked by the implementation of a screening question aimed at evaluating the engagement of the child in video game activity during the school closure period. Those students who did not partake in video game activity were subsequently excluded from this study, and consequently, their parents did not proceed to participate in the interview process.

### Sample size calculation

To calculate the sample size, we used the following formula:

$$n\, = \,\left[ {Z^2 p \, x \, \left( {1 - p} \right)} \right] \, / \, d^2 ,$$

where *n* represents the sample size. *P* is the prevalence from previous literature, and according to our literature review the prevalence IGD during the COVID-19 pandemic in other countries was 5% [19]. *d* is the precision or margin of error which was set at 3% (0.03). *Z* is the Z statistic for an α error of 0.05 corresponding to a 95% confidence level, which is equal to 1.96. The calculated sample size is 203.

To account for the low response rate encountered in telephone interviewes, which we expected to be about 30% based on our experience in a previous study we conducted, we approached a total of 700 parents.

### The data collection tool and outcome measures

The questionnaire was adapted from other validated tools [20, 21]. The first section of the questionnaire addressed the sociodemographic characteristics of the students and their parents. The second part assessed the screen time (time spent using any type of digital devices like smartphones, laptops, computers, Tablets, TV excluding time spent in online classes) and the duration of playing video games (including both online and offline games, on any device (such as consoles, computers, tablets, or smartphones) during weekdays and weekends, in two periods (prior to and during school closures) [20]. The weekly screen and video gaming times for each student were calculated by multiplying the weekday times by 5 and the weekend times by 2 and then summing the two values. The change in gaming time was calculated by subtracting the weekly gaming time before from weekly gaming time during school closures. Positive values were labeled as increased video gaming time. The third section is the parental version of Internet Gaming Disorder Scale (PIGDS), which is a nine items dichotomous scale that was adapted from the adolescent’s version of Internet Gaming Disorder Scale by Lutz Wartberg et al. and showed an internal consistency of 0.86 and excellent validity [21]. The dichotomous response format of the original IGDS (0 = ‘‘no,’’ 1 = ‘‘yes’’) was maintained for the PIGDS. By summing up the values of all nine items of the instrument, a PIGDS sum score was computed with a higher sum indicating higher risk levels of IGD. Those with scores of 5 or more were classified as having IGD [21]. The final questionnaire was translated into Arabic. To ensure the translational validity of our instrument, we employed a rigorous translation and back translation process. Initially, the instrument was translated from English to Arabic by independent translators proficient in both English and Arabic. Following this, a third individual, who was not privy to the original English version, conducted a back translation of the instrument into English. This back-translated version was then meticulously compared with the original English version to identify and rectify any inconsistencies or discrepancies. Subsequent adjustments were made to the Arabic version to enhance its accuracy and fidelity to the original. Furthermore, we conducted a pilot test of the Arabic version with a group of native Arabic speakers. Their feedback, particularly regarding comprehension and clarity, was instrumental in refining the instrument. Any necessary modifications were incorporated based on this feedback. The finalized versions of the questionnaire, in both English and Arabic, are available in the supplementary materials (Supplementary Materials 1 and 2).

### Data analysis

The statistical analysis was performed using IBM SPSS (Statistical Product and Service Solutions) software for Windows, Version 26.0 (IBM Corp, Armonk, NY). We utilized percentages to summarize categorical data and mean and standard deviation for numerical data. Normality was assessed using the Shapiro–Wilk test. Univariable analysis was carried out using appropriate tests such as Chi-square or Fisher's exact test for categorical variables, and the Mann–Whitney *U* test for comparing the change in video gaming time across groups.

To determine the significance of changes in gaming time over the period of school closures, we employed the Wilcoxon Singed Rank test. Logistic regression analysis was used to explore potential predictors of developing IGD. The selection of variables for the regression model in our study was guided by a combination of statistical criteria and a comprehensive review of relevant literature. Specifically, we included variables in the model that demonstrated a *p* value of less than 0.25 in the bivariate analysis, indicating their potential significance. Additionally, our choices were informed by insights and findings from existing literature, ensuring that the variables selected were not only statistically relevant but also contextually pertinent to our research objectives. The associations between risk factors and outcomes were presented as adjusted Odds Ratios (AOR) and 95% confidence intervals (95% CIs). Statistical significance was set at a *P* value less than 0.05.

### Ethical considerations

This study was ethically approved by Primary Health Care Corporation review board.

To ensure the confidentiality and privacy of the data collected in this study, we implemented several rigorous measures. All data were collected de-identified, with participants assigned unique identifiers to maintain their anonymity. This approach ensured that personal information such as names or other direct identifiers were not attached to the responses. Furthermore, the collected data were used strictly for research purposes only. To safeguard this sensitive information, it was stored on a password-protected computer, accessible only to the research team. Prior to data collection, all data collectors underwent comprehensive training on handling sensitive information and maintaining participant confidentiality. Additionally, they signed confidentiality agreements to legally bind them to uphold the privacy and security of the data. These security measures were in place to prevent unauthorized access and to ensure that the integrity and confidentiality of the data were maintained throughout the course of the research.

## Results

### Sociodemographic and background information

A total of 700 parents were approached by phone, out of whom, 428 answered the screening question, and 257 (60% of the 428) confirmed their child’s engagement in video gaming during the period of school closures giving an overall response rate of 36.7% (257/700). The study's student participants exhibited an average age of 11 years (11 ± 2 SD), with 74.3% falling within the 8–11 years age category and 25.7% between 12 and 15 years. The gender distribution comprised 92 (35.8%) females and 165 (64.2%) males. In terms of nationality, 62.6% were expatriates, and the remaining were Qatari locals. Family sizes varied, with approximately 41% having three or fewer siblings, and 59% having more than three. A history of visual disturbances was reported by 14.8% of students. Parents' ages averaged 39 years for mothers and 45 years for fathers. Maternal education revealed 46.3% with college degrees or higher. Furthermore, 45.5% of mothers were employed, while 54.5% were not (Table 1).

**Table 1:** Sociodemographic characteristics of included students and background information

Characteristic	no. (%)
Student age (M ± SD)	11 ± 2
Student’s age category	
8–11 years	191 (74.3)
12–15 years	66 (25.7)
Gender	
Female	92 (35.8)
Male	165 (64.2)
Nationality	
Expatriates	161 (62.6)
Locals (Qatari)	96 (37.4)
Number of siblings	
3 or less	105 (40.9)
> 3	152 (59.1)
History of visual disturbances	
No	219 (85.2)
Yes	38 (14.8)
Mother’s age (M ± SD)	39 ± 6
Father’s age (M ± SD)	45 ± 7
Mother’s education	
No formal education	5 (1.9)
Secondary/high school level	133 (51.8)
College or higher	119 (46.3)
Father’s education	
No. formal education	4 (1.6)
School level education	114 (44.4)
College or higher	139 (54.1)
Mother’s employment status	
Employed	117 (45.5)
Not employed	140 (54.5)

*M*, Mean; *SD*, Standard deviation

### The changes in screen and video gaming times over the period of school closures

Before the school closure, students in the total sample reported an average screen time of 19.7 ± 10.1 h per week which increased significantly to 31.9 ± 12.6 h per week during the closure (Table 2). A similar pattern was observed in screen time on weekdays, rising from 2.6 ± 1.5 h per day to 4.5 ± 1.8 h per day during the closure. On weekends, the corresponding values were 3.4 ± 1.9 h per day before closure and 4.7 ± 2.1 h per day during closure. The observed changes were significant with *p* < 0.001.

**Table 2:** Screen and video gaming times before and during school closures in the total sample and in different subgroups

Variables	Total sample M ± SD	Not meeting the diagnostic cut point for Internet Gaming Disorder M ± SD	Mean Rank	Meeting the diagnostic cut point for Internet Gaming Disorder M ± SD	Mean Rank	P value*
*Screen time (in hours)*						
All week (hours/week)						
Before school closure	19.7 ± 10.1	20.1 ± 10.1	132.1	15.7 ± 9.8	95.6	0.027
During school closure	31.9 ± 12.6	31.4 ± 12.3	126.0	38.1 ± 14.2	161.5	0.031
On weekdays (hours/day)						
Before school closure	2.6 ± 1.5	2.7 ± 1.5	134.2	1.6 ± 1.4	72.2	< 0.001
During school closure	4.5 ± 1.8	4.4 ± 1.8	127.4	5.1 ± 2.3	145.8	0.261
On weekends (hours/day)						
Before school closure	3.4 ± 1.9	3.3 ± 1.8	127.3	4.0 ± 2.6	146.7	0.235
During school closure	4.7 ± 2.1	4.6 ± 2.0	124.0	6.4 ± 2.4	182.0	< 0.001
*Video gaming time (in hours)*						
All week (hours/week)						
Before school closure	8.6 ± 8.6	8.1 ± 8.3	125.8	13.5 ± 10.9	163.4	0.020
During school closure	13.0 ± 12.4	11.5 ± 11.1	121.6	28.9 ± 14.4	207.6	< 0.001
On weekdays (hours/day)						
Before school closure	1.1 ± 1.2	1.1 ± 1.2	127.4	1.5 ± 1.5	146.3	0.229
During school closure	1.8 ± 1.7	1.6 ± 1.6	121.7	4.0 ± 2.0	207.2	< 0.001
On weekends (hours/day)						
Before school closure	1.5 ± 1.6	1.4 ± 1.4	124.6	3.1 ± 2.5	175.9	0.001
During school closure	2.0 ± 1.9	1.8 ± 1.7	121.9	4.5 ± 2.5	204.7	< 0.001

*M*, Mean; *SD*, Standard deviation

^*^ Using Mann–Whitney test

**Fig. 1:**
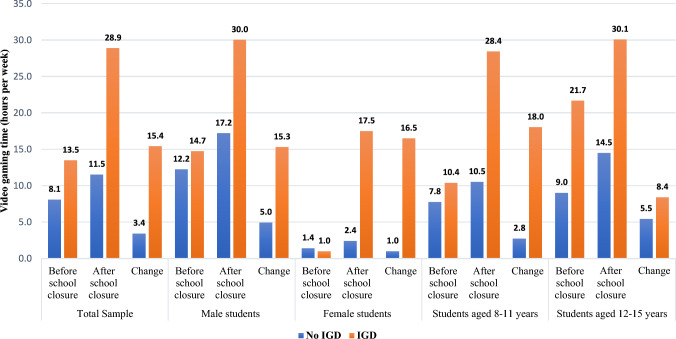
Video gaming time before and during school closures in the total sample, both sexes and age groups by gaming addiction status. *IGD*; Internet Gaming Disorder

Concerning video gaming time, the total sample reported spending 8.6 ± 8.6 h per week on video games before the closure, which increased significantly to 13.0 ± 12.4 h per week during the closure (*p* < 0.001) as shown in Fig. 1. The daily gaming hours followed a similar trend: 1.1 ± 1.2 h per day before closure and 1.8 ± 1.7 h per day during closure on weekdays, and 1.5 ± 1.6 h per day before closure and 2.0 ± 1.9 h per day during closure on weekends as shown in Table 2.

In terms of screen time changes, the total sample reported an average change of 12.2 ± 11.2 h per week (equivalent to an increase of 1.7 ± 1.6 h/day). When stratified by IGD status, individuals not meeting the criteria for IGD exhibited a change of 11.3 ± 10.2 h per week, whereas those meeting the IGD criteria showed a substantial increase with a mean change of 22.3 ± 16.3 h per week. These differences were statistically significant (*p* = 0.001), indicating that the change in screen time was notably higher among those meeting the diagnostic cut point for IGD.

Similarly, significant differences were observed in the changes in video gaming time between the two groups. The total sample reported an average change of 4.5 ± 7.9 h per week (equivalent to an increase of 0.6 ± 1.1 per day). Among individuals not meeting the IGD criteria, the mean change was 3.4 ± 5.9 h per week. In contrast, those meeting the IGD criteria exhibited a larger change with a mean of 15.4 ± 15.5 h per week (Fig. 1). These differences were highly statistically significant (*p* < 0.001), underscoring the pronounced change in video gaming time among individuals meeting the IGD criteria. For changes in both screen time and video gaming time, the differences between the two groups were consistently evident across various timeframes (all week, weekdays, and weekends) and were statistically significant (Table 3).

**Table 3:** Comparing the change of screen and video gaming times across the addicted and non-addicted groups

Variables	Total sample	Not meeting the diagnostic cut point for Internet Gaming Disorder M ± SD	Mean Rank	Meeting the diagnostic cut point for Internet Gaming Disorder M ± SD	Mean rank	*P* value*
Screen time (in hours)						
Change all week (hours/week)	12.2 ± 11.2	11.3 ± 10.2	124.4	22.3 ± 16.3	177.9	**0.001**
Change on weekdays (hours/day)	1.9 ± 1.7	1.7 ± 1.6	124.4	3.5 ± 2.5	178.3	**0.039**
Change on weekends (hours/day)	1.4 ± 1.7	1.3 ± 1.5	126.2	2.4 ± 2.4	159.0	**0.001**
Video gaming time (in hours)						
Change all week (hours/week)	4.5 ± 7.9	3.4 ± 5.9	122.9	15.4 ± 15.5	194.6	** < 0.001**
Change on weekdays (hours/day)	0.7 ± 1.2	0.5 ± 0.9	122.6	2.5 ± 2.3	197.6	** < 0.001**
Change on weekends (hours/day)	0.5 ± 1.1	0.4 ± 0.8	126.3	1.5 ± 2.4	157.5	**0.018**

Significant *p* values are bolded

*M*, Mean; *SD*, Standard deviation

^*^ Using Mann–Whitney test

### Prevalence of internet gaming disorder among students in Qatar and associated factors

The prevalence of internet gaming disorder in our sample among those aged 8–15 years was found to be 8.6% (22/257) with 95% CI (5.4–12.7). The prevalence was significantly higher among male students, expatriates, and those with small family size (3 siblings or less) as shown in Table 4. Male students had significantly higher percentages of IGD compared to females (12.1% vs 2.2%, *p* = 0.006), while no significant difference was found across age groups (Table 4). The logistic regression analysis showed that the nationality (Expatriate) and increasing video gaming times as significant predictors of developing IGD during the period of school closures. Expatriate students were approximately 14 times more likely to develop IGD as compared to their Qatari peers (AOR 14.11, 95% CI 1.69–117.89, *p* = 0.015). Similarly, students who reported an increase in video gaming times were 6.7 times more likely to develop IGD than those who did not (AOR 6.78, 95% CI 1.65–27.81, *p* = 0.008), see Table 4.

**Table 4:** Determinants and predictors of developing Internet Gaming Disorder among students during school closures in Qatar

Characteristic	Meeting the diagnostic cut point for Internet Gaming Disorder		*P* value*	Multivariable logistic regression	
	No	Yes			
	No (%)	No (%)		AOR (95%CI)	*P* value
Student’s age category					
8–11 years	175 (91.6)	16 (8.4)	0.858	[Reference]	
12–15 years	60 (90.9)	6 (9.1)		1.34 (0.35–5.20)	0.673
Gender					
Female	90 (97.8)	2 (2.2)	**0.006**	[Reference]	
Male	145 (87.9)	20 (12.1)		2.65 (0.51–13.80)	0.248
Nationality					
Locals (Qatari)	94 (97.9)	2 (2.1)	**0.004**	[Reference]	
Expatriates	141 (87.6)	20 (12.4)		14.11 (1.69–117.89)	**0.015**
Number of siblings					
3 or less	91 (86.7)	14 (13.3)	**0.023**	[Reference]	
> 3	144 (94.7)	8 (5.3)		1.86 (0.55–6.29)	0.316
History of visual disturbances					
No	198 (90.4)	21 (9.6)	0.216	[Reference]	
Yes	37 (97.4)	1 (2.6)		2.94 (0.32–26.85)	0.340
Mother’s age (M ± SD)	39 ± 6	37 ± 5	0.188	0.96 (0.83–1.10)	0.546
Father’s age (M ± SD)	45 ± 7	44 ± 6	0.453	1.04 (0.93–1.16)	0.526
Mother’s education					
No formal education	3 (60.0)	2 (40.0)	**0.039**	[Reference]	
Secondary/high school level	125 (94.0)	8 (6.0)		2.32 (0.11–50.24)	0.591
College or higher	107 (89.9)	12 (10.1)		0.57 (0.10–3.09)	0.512
Father’s education					
No formal education	3 (75.0)	1 (25.0)	**0.036**	[Reference]	
School level education	104 (91.2)	10 (8.8)		16.77 (0.38–745.01)	0.145
College or higher	128 (92.1)	11 (7.9)		2.44 (0.50–11.93)	0.270
Mother’s employment status					
Employed	106 (90.6)	11 (9.4)	0.659	[Reference]	
Not employed	129 (92.1)	11 (7.9)		1.10 (0.33–3.66)	0.880
Change in video gaming time					
Stayed the same or decreased	147 (96.7)	5 (3.3)	** < 0.001**	[Reference]	
Increased	88 (83.8)	17 (16.2)		6.78 (1.65–27.81)	**0.008**

Significant *p* values are bolded

*AOR*, Adjusted odds ratio; *CI*, Confidence interval

*Bivariate analysis using Chi-square or Fisher’s Exact tests

## Discussion

The mitigation of COVID-19's transmission prompted nations to implement closures of educational establishments, including schools. Consequently, the conventional in-person instructional techniques underwent a transformation into virtual online education, necessitating extended durations of screen exposure. Concurrently, children and adolescents increasingly turned to digital devices for recreational and social purposes [22]. This surge in screen time has significant adverse implications for the physical and mental well-being of young individuals. These repercussions encompass disruptions in sleep patterns and psychological equilibrium, an escalation in sedentary behaviors coupled with diminished physical activity, and a potential detrimental impact on ocular health [23]. Comparatively, the extent of the rise in non-academic screen time in our study appeared to surpass that reported in some studies conducted in Singapore, the USA, and China [24]. This discrepancy could be attributed to a range of factors, including variations in educational policies, cultural attitudes toward technology, and the availability of digital infrastructure. Moreover, the dynamic nature of the pandemic, including fluctuating infection rates and the evolving impact on daily life, adds an additional layer of complexity to the comparative analysis.

The imposition of home confinement measures, including the closure of educational institutions, has directed the focus of children toward indoor pursuits, particularly the engagement in electronic video gaming. In the present study, a noteworthy escalation in the duration spent on video gaming was observed during the period of closure, supporting the findings of previous studies [19, 25]. Early in the course of the pandemic, the hashtag “Play Apart Together” garnered widespread attention, gaining prominence as part of a campaign endorsed by the World Health Organization (WHO) in collaboration with major gaming industries. This initiative encouraged video gaming to foster social connections while adhering to physical distancing recommendations [26]. Notably, discussions emerged around whether this campaign signaled a change in WHO's perspective on gaming disorders. More recently, the WHO's “Healthy At Home” campaign highlighted the significance of maintaining an appropriate balance between online activities, including video games, and offline pursuits [27]. Despite the potential merits associated with video gaming, excessive gaming is time consuming and has deleterious implications for health. A new term “videogame vision syndrome” has been introduced to specifically address vision issues related to long periods of continuous use of screen-enabled devices to play videogames [17].

The observed prevalence of 8.6% indicates that an unignorable portion of students aged 8–15 years within the sample meets the criteria for IGD. This figure emphasizes the significance of IGD as a pertinent issue affecting a considerable segment of children and adolescents. This finding aligns with prior research studies that have highlighted the growing concern of excessive internet-based activities, particularly in the context of gaming, among adolescents [28, 29]. However, it is crucial to approach these findings with caution due to the methodological variability in assessing gaming addiction.

Our findings indicate a higher propensity for the development of Internet Gaming Disorder (IGD) among male students in comparison to their female counterparts, aligning with established literature on the subject. This observation supports the notion that gender may play a role in susceptibility to IGD [30, 31]. However, it is important to note that this gender difference was not observed in our multivariate regression analysis, indicating that when controlling for other variables, the direct effect of gender on IGD may be less pronounced or potentially mediated by other factors. On the other hand, male gender was found as a significant predictor in other studies [32]. The greater likelihood of males developing gaming addiction in comparison to females observed in the literature can be partially attributed to gender-related variations in neural responses to cravings, a significant precursor to addictive behaviors. Previous research has revealed that neural activations linked to cravings, such as in the insula and dorsolateral prefrontal cortex, show gender-specific differences, that are particularly evident in substance addictions like cocaine and gambling, suggesting distinct underlying mechanisms that might contribute to addictive tendencies in males and females [33]. This trend could also be elucidated by considering the prevalent competitive nature of video games, which tends to be more appealing to boys than girls [34].

Remarkably, expatriate students demonstrated a higher likelihood of developing Internet Gaming Disorder (IGD) in comparison to their local Qatari counterparts in this study. This contrast could be attributed to several factors, including potential differences in cultural backgrounds, social support systems, and coping mechanisms. Expatriate students often come from diverse cultural backgrounds, which may influence their leisure activities and coping strategies. The role of video games in their home cultures, along with varying norms and attitudes toward gaming, could contribute to higher engagement in gaming as a familiar and accessible form of entertainment in a foreign setting. Moreover, the transition to a new country often disrupts traditional social networks, leaving expatriate students more reliant on virtual communities for social interaction. Online gaming platforms can offer a sense of community and belonging, potentially leading to increased gaming time as a substitute for real-world social interactions. It is expected that expatriates may face unique stressors such as homesickness, cultural adjustment challenges, and academic pressures in an unfamiliar environment. Gaming might serve as an escape mechanism, providing relief from these stressors but also increasing the risk of developing IGD. Additionally, the language barrier, a significant challenge for many expatriates, may have been less of an impediment in the gaming context, further encouraging engagement in online gaming activities. The lack of direct family supervision or guidance, particularly for those expatriates living away from their families, might have also contributed to less regulated gaming habits. Expatriate students may face challenges such as feelings of isolation due to being away from their home country particularly in the setting of travel restrictions imposed during the pandemic, leading them to turn to online gaming as a means of connection and stress relief. Further research is warranted to thoroughly investigate the underlying dynamics driving this divergence in IGD susceptibility between expatriate and local students.

A recent meta-analysis conducted globally demonstrated an inverse linkage between IGD and individuals' emotional well-being [35]. These findings suggest that individuals susceptible to IGD tend to exhibit heightened levels of loneliness, sleep disturbances, and compromised concentration [36]. Furthermore, such individuals are more likely to engage in aggressive behaviors, display impulsivity, and demonstrate inclinations toward self-harm [36]. While these observations are globally recognized, their implications in the local and regional context of our study, particularly in the Middle East, warrant further discussion.

In our region, the unique social, cultural, and economic dynamics may exacerbate these mental health challenges associated with IGD. Factors such as social isolation, which can be intensified by cultural integration challenges faced by expatriate populations, and the rapid pace of modernization impacting traditional social structures, are likely to contribute to the heightened levels of loneliness and sleep disturbances observed [37]. Additionally, regional differences in the perception and handling of mental health issues could influence the manifestation and recognition of symptoms like aggression and impulsivity, which are associated with IGD [38].

The impact of these regional factors is particularly pertinent considering the backdrop of the COVID-19 pandemic, which has further amplified issues of social isolation and disrupted traditional support systems. This context provides a critical lens through which to interpret the global findings on IGD and emotional well-being, stressing upon the need for region-specific strategies in addressing these challenges.

In order to address the issue of IGD, it is imperative to adopt a multifaceted approach involving various stakeholders, as suggested by the WHO [39]. Parents and guardians, as primary caregivers, should empower children with information on safe online practices and encourage a balanced blend of online and offline activities. In Bangkok, a school and family-based intervention program significantly reduced gaming addiction in 4th and 5th graders, with an 8-week curriculum focusing on self-regulation, parental guidance, and teacher training [40]. Clear rules and boundaries regarding screen time usage should be established, alongside the installation of appropriate software safeguards on devices and the encouragement of physically active gaming experiences. A recent study evaluating apps designed to reduce mobile phone use demonstrated that features like self-tracking, goal setting, and usage limits were effective in managing phone usage and preventing maladaptive behaviors [41]. Health and social care providers play a crucial role in disseminating information about the risks associated with excessive screen time or gaming to families. Regular assessments should incorporate considerations of screen time and video gaming habits, alongside the provision of psychological support and counseling for those dealing with gaming disorders.

Digital technology and gaming companies can contribute by incorporating safety features and parental controls into their products. Furthermore, they should consider the development of gaming programs that encourage physical activity, thereby promoting healthier gaming habits. The use of active video games, or exergaming, can effectively enhance physical activity in adolescents, offering a more acceptable and sustainable alternative to traditional methods [42]. Moreover, it can reduce both the body mass index percentile and total cholesterol in overweight and obese adolescents [43]. By prioritizing balanced screen time, implementing protective measures, and offering psychological support, a comprehensive approach can mitigate the negative consequences of excessive online gaming, thereby fostering healthier digital engagement and overall well-being among children and adolescents.

## Strengths and limitations

This study contributes novel insights into the effects of COVID-19-induced school closures on the screen time and video gaming behaviors of governmental school students. Notably, this research is the first of its kind conducted in Qatar and among the few regionally to investigate this area comprehensively. By focusing on an important segment of the population—the student cohort—our study addresses a critical gap in literature. The utilization of standardized measurements, such as the Internet Gaming Disorder scale, bolsters the reliability and comparability of our results. However, it is essential to acknowledge the limitations inherent in our study. The retrospective data collection method, reliant on parental reporting, introduces the potential for recall bias, possibly affecting the accuracy of reported screen time and gaming behaviors. The reliance on self-reported measures might not capture the full extent of the problem due to underreporting or social desirability effects. A notable limitation of our study is the lack of distinction between different gaming platforms and between online and offline games while assessing IGD. The experiences and potential risks associated with IGD might vary not only across different platforms, such as consoles, computers, and mobile devices, but also between online and offline gaming environments. Each platform and mode of gaming has unique characteristics and usage patterns that could influence the development and manifestation of IGD in distinct ways. However, due to the scope and design of the current study, we were unable to explore these specific differences. We believe that these aspects of IGD, including the differences between online and offline gaming as well as between various gaming platforms, warrant a more detailed investigation. Such focused research could provide valuable insights into tailored prevention and intervention strategies for IGD, considering the diverse gaming environments and experiences.

## Conclusion

This study examined the implications of the school closures imposed during the COVID-19 pandemic on screen time and video gaming behaviors of governmental school students aged 8–15 years in Qatar. Significant increases in the screen and video gaming times were observed. The study revealed an IGD prevalence of 8.6% within our sampled population. These outcomes highlights the profound impact of pandemic-related disruptions on the digital engagement patterns of students and raise pertinent concerns regarding the potential escalation of IGD in this context. Further research is crucial to comprehensively understand the long-term consequences of extended screen time and gaming habits in the wake of the pandemic.

## Supplementary Information

Below is the link to the electronic supplementary material.

Supplementary file1 (DOCX 35 KB)

Supplementary file2 (DOCX 36 KB)

## Data Availability

Data will be made available upon reasonable request from the corresponding author.
