# Supplementary material for: From classrooms to controllers: how school closures shaped children's video gaming habits
Source: Soc Psychiatry Psychiatr Epidemiol. 2024 Mar 12;59(12):2259–69. doi: 10.1007/s00127-024-02635-z (PMC11522056; doi:10.1007/s00127-024-02635-z)
Supplement: Supplementary file 1 — Supplementary file1 (DOCX 35 KB) [file 127_2024_2635_MOESM1_ESM.docx]

**Supplementary Material 1: The English version of the questionnaire**

| **Screening question:** Did your child use to play video games (including both online and offline games, on any device (such as consoles, computers, tablets, or smartphones) during the period of COVID-19 related school closures? Yes or No (if yes complete the questionnaire) | | | | | | | |
| --- | --- | --- | --- | --- | --- | --- | --- |
| **Section A**: **Socio-demographic characteristics and background information:** | | | | | | | |
| **Question** | **Choices:** | | | | | | |
| 1. Age |  | | | | | | |
| 1. Age of the mother (in years) |  | | | | | | |
| 1. Age of the father (in years) |  | | | | | | |
| 1. Nationality of the child |  | | | | | | |
| 1. Highest degree of education for mother? | No formal education | Secondary/High school diploma | | | College or Higher | | |
| 1. Highest degree of education for Father? | No formal education | Secondary/High school diploma | | | College or Higher | | |
| 1. Number of child siblings |  | | | | | | |
| 1. Mother employment status | Employed | | Not employed | | | | |
| 1. Total family income per month (in QAR) | Less than 10000 | | 10000-30000 | | | 30000-50000 | More than 50000 |
| 1. has your child ever been diagnosed with any eye problem or disease | Yes (specify) | | | No | | | |

| **Section B: Duration of digital device use before and during school closures** | | |
| --- | --- | --- |
| 1. How many hours did your child use to use digital devices in a day (excluding time spent for online school classes)? *Before school closure* | During Weekdays |  |
|  | During Weekend |  |
| 1. How many hours did your child use to use digital devices in a day (excluding time spent for online school classes)? *During school closure* | During Weekdays |  |
|  | During Weekend |  |
| 1. How many hours did your child use to play video games including both online and offline games, on any device (such as consoles, computers, tablets, or smartphones) in a day *Before school closure* | During Weekdays |  |
| 1. How many hours did your child use to play video games including both online and offline games, on any device (such as consoles, computers, tablets, or smartphones) in a day *During school closure* | During Weekend |  |
| **Section C: PIGDS** | | |
| 1. During the past 12 months indicate whether you noticed the following on your child | | |
| Have there been periods when all your child could think of was the moment that she/he could play a game? | Yes | No |
| Has your child felt dissatisfied because she/he wanted to play more? | Yes | No |
| has your child been feeling miserable when she/he was unable to play a game? | Yes | No |
| was your child unable to reduce her/his time playing games, after others had repeatedly told her/him to play less? | Yes | No |
| Has your child played games so that she/he would not have to think about annoying things? | Yes | No |
| has your child had arguments with others about the consequences of her/his gaming behavior? | Yes | No |
| Has your child hidden the time she/he spends on games from others? | Yes | No |
| has your child lost interest in hobbies or other activities because gaming is all she/he wanted to do? | Yes | No |
| has your child experienced serious conflicts with family, friends, or partner because of gaming? | Yes | No |
