# Supplementary material for: From classrooms to controllers: how school closures shaped children's video gaming habits
Source: Soc Psychiatry Psychiatr Epidemiol. 2024 Mar 12;59(12):2259–69. doi: 10.1007/s00127-024-02635-z (PMC11522056; doi:10.1007/s00127-024-02635-z)
Supplement: Supplementary file 2 — Supplementary file2 (DOCX 36 KB) [file 127_2024_2635_MOESM2_ESM.docx]

**Supplementary Material 2: The Arabic version of the questionnaire**

| **سؤال مسحي: هل اعتاد طفلك على لعب ألعاب الفيديو بما في ذلك الألعاب عبر الإنترنت وغير المتصلة بالإنترنت، على أي جهاز (مثل وحدات التحكم أو أجهزة الكمبيوتر أو الأجهزة اللوحية أو الهواتف الذكية)**  **اذا لا سيتم استبعاد الطالب من البحث** | | | | | | |
| --- | --- | --- | --- | --- | --- | --- |
| **القسم أ: الخصائص الاجتماعية والديموغر افية ومعلومات أساسية :** | | | | | | |
| **الخيارات:** | | | | | | **السؤا ل** |
|  | | | | | | 1. العمر |
|  | | | | | | 1. عمر الأم(بالسنوات) |
|  | | | | | | 1. عمر الأب(بالسنوات) |
|  | | | | | | 1. جنسية الطفل |
| شهادة جامعية أو أعلى | | شهادة الثانوية | | لا يوجد تعليم رسمي | | 1. ما هو أعلى مستوى تعليمي للأم؟ |
| شهادة جامعية أو أعلى | | شهادة الثانوية | | لا يوجد تعليم رسمي | | 1. ما هو أعلى مستوى تعليمي للأب؟ |
|  | | | | | | 1. عدد أشقاء الطفل |
| غير موظفة | | | موظفة | | | 1. ما هي حالة توظيف الأم؟ |
| أكثر من 50,000 | 50,000 – 30,000 | | 30,000 - 10,000 | | أقل من 10,000 | 1. الدخل الإجمالي الشهري للأسرة (بالريال القطري) |
| لا | | | نعم (حدد) | | | 1. هل سبق أن تم تشخيص طفلك بأي مشكلة أو مرض في العين؟ |

| **القسم د: مدة استخدام الأجهزة الرقمية قبل وأثناء إغلاق المدارس** | | |
| --- | --- | --- |
|  | خلال أيام الأسبوع | 1. ما إجمالي عدد الساعات التي اعتاد طفلك أن يقضيها في استخدام الأجهزة الرقمية في اليوم (باستثناء الوقت المخصص للتعليم عن بعد) قبل إغلاق المدرسة ؟ |
|  | خلال عطلة نهاية الأسبوع |  |
|  | خلال أيام الأسبوع | 1. ما إجمالي عدد الساعات التي اعتاد طفلك أن يقضيها في استخدام الأجهزة الرقمية في اليوم (باستثناء الوقت المخصص للتعليم عن بعد (قبل إغلاق المدرسة ؟ |
|  | خلال عطلة نهاية الأسبوع |  |
|  | خلال أيام الأسبوع | 1. كم ساعة اعتاد طفلك أن يلعب ألعاب الفيديو في اليوم بما في ذلك الألعاب عبر الإنترنت وغير المتصلة بالإنترنت، على أي جهاز (مثل وحدات التحكم أو أجهزة الكمبيوتر أو الأجهزة اللوحية أو الهواتف الذكية) قبل إغلاق المدرسة؟ |
|  | خلال عطلة نهاية الأسبوع | 1. كم ساعة اعتاد طفلك يلعب ألعاب الفيديو في اليوم بما في ذلك الألعاب عبر الإنترنت وغير المتصلة بالإنترنت، على أي جهاز (مثل وحدات التحكم أو أجهزة الكمبيوتر أو الأجهزة اللوحية أو الهواتف الذكية) أثناء إغلاق المدرسة؟ |
| **القسم ه: استبيان اضطراب العاب الفيديو** | | |
| 1. **خلال ال 12 شهر السابقة هل لاحظت على طفلك ما يلي:** | | |
|  |  | هل مرت اوقات كل ما يفكر فيه طفلك هو الموعد الذي يستطيع فيه االلعب بألعاب الفيديو؟ |
|  |  | هل طفلك يشعر بأنه غير راضي لانه يود اللعب اكثر بألعاب الفيديو ؟ |
|  |  | هل يشعر طفلك بالتعاسة عندما لا يستطيع ان يلعب ألعاب الفيديو؟ |
|  |  | هل طفلك لا يستطيع ان يقلل اوقات استخدامه لالعاب الفيديو بعدما اخبره الاخرين بأن عليه تقليل وقت اللعب؟ |
|  |  | هل يلعب طفلك العاب الفيديو حتى لا يفكر بأمور تزعجه؟ |
|  |  | هل حدث مع طفلك جدالات مع الاخرين لرغبته باللعب بالرغم من معرفته بعواقب سلوكه في اللعب؟ |
|  |  | هل يخفي طفلك عن الاخرين الوقت الذي يقضيه في العاب الفيديو؟ |
|  |  | هل فقد طفلك الاهنمام بالهوايات او الانشطة الأخرى لأن اللعب بألعاب الفيديو هو كل ما يريد القيام به؟ |
|  |  | هل عانى طفلك من خلافات حادة مع العائلة او الاصدقاء بسبب اللعب بألعاب الفيديو؟ |
